# Voting Charities

**Published:** 1891-04-25

**Authors:** 


### Passing Topics.

### VOTING CHARITIES.

Our old friend, the voting charity, has again been very much in evidence. That doughty opponent of the voting system, Mr. F. D. Mocatta, has once more led an attack upon it. Once more he has scored all the old successes, so far as mere argument is concerned, and once more he must be content with a barren glory—for the results are practically *nil*.

The champions of the system hold their ground obstinately, and apparently are not wanting in earnestness. They resent every suggestion of reform, and advance propositions which we are bound to believe they honestly believe in, however little their dialectical skill is capable of supporting them, and however little, if supported, these contentions would bear upon the real point at issue. The fact is there is but one excuse for the voting system, and it is that it brings in subscriptions. That it does this is undoubted and undoubtable. But in these preternaturally enlightened times we ought not to need to be convinced that there is more to be aimed at than to conserve the interests of the "institution" as apart from the interests of those for whom the institution exists.

The voting system is obviously and notoriously a cruel system. The benefits obtained by its means are only reached through a painful ordeal. To those who need them most they are most inaccessible, and if ever anybody has known what it is to be sick with hope deferred, it must be the friendless candidate upon the list of a voting cum-canvassing charity, whose scanty means and too often scanty remnant of life are alike consumed in feverish struggles to enlist the suffrages of a legion of subscribers.

We only know of one system of torture applied in the name of charity which compares with it, and that is in connection with those societies for supplying surgical instruments, which demand of the sufferer and his scarcely less unhappy friends, the collection of "letters" sufficient, at the value of a subscription each, to cover the cost of the particular instrument the surgeon has prescribed as necessary. And in the judgment of humanity, the surgical society would have the advantage, for it does not demand a palpably impossible task, which at best must take many months, or even years, to complete, while little by little all the victim knows of hope and strength and peace melts, until one can almost picture him turning away to die with a curse in his heart.

If the upholders of the canvassing system think this description overdrawn, let them try to put themselves in the place of one of these unfortunates condemned to wracking suspense, and all the worry, labour, and outlay which the position of an accepted candidate involves. The very first requisite of charitable relief, if it is to be a reality, is that it shall be promptly rendered. Under the voting system, as it is practised in connection with several of our greatest institutions, we see the utmost delay and difficulty interposed between the applicant and the coveted assistance. The organisation which claims to minister to the needy traverses its own pretensions by exacting a terrible and prepaid price for its favours. The so-called field of charity must be cultivated by the sufferers' enfeebled hands, and to make the soil prolific of subscriptions it must be watered with their blood.

It is inevitable that a purely voluntary provision should often prove inadequate to all that is demanded of it, but we would fain hope that the ingenuity of professional philanthropy will never prove capable of devising another such engine of cruelty and extortion as the voting-cum-canvassing system. Agricola.

